# Optimal functional outcome measures for assessing treatment for Dupuytren’s disease: a systematic review and recommendations for future practice

**DOI:** 10.1186/1471-2474-14-131

**Published:** 2013-04-10

**Authors:** Catherine Ball, Anna L Pratt, Jagdeep Nanchahal

**Affiliations:** 1 Kennedy Institute, University of Oxford, Aspenlea Road, London W6 8LH, UK; 2 School of Health Sciences and Social Care, Brunel University, Kingston Lane, Uxbridge, UB8 3PH, UK

**Keywords:** Dupuytren’s disease, Hand function, Outcome measures, Systematic review

## Abstract

**Background:**

Dupuytren's disease of the hand is a common condition affecting the palmar fascia, resulting in progressive flexion deformities of the digits and hence limitation of hand function. The optimal treatment remains unclear as outcomes studies have used a variety of measures for assessment.

**Methods:**

A literature search was performed for all publications describing surgical treatment, percutaneous needle aponeurotomy or collagenase injection for primary or recurrent Dupuytren’s disease where outcomes had been monitored using functional measures.

**Results:**

Ninety-one studies met the inclusion criteria. Twenty-two studies reported outcomes using patient reported outcome measures (PROMs) ranging from validated questionnaires to self-reported measures for return to work and self-rated disability. The Disability of Arm, Shoulder and Hand (DASH) score was the most utilised patient-reported function measure (n=11). Patient satisfaction was reported by eighteen studies but no single method was used consistently. Range of movement was the most frequent physical measure and was reported in all 91 studies. However, the methods of measurement and reporting varied, with seventeen different techniques being used. Other physical measures included grip and pinch strength and sensibility, again with variations in measurement protocols. The mean follow-up time ranged from 2 weeks to 17 years.

**Conclusions:**

There is little consistency in the reporting of outcomes for interventions in patients with Dupuytren’s disease, making it impossible to compare the efficacy of different treatment modalities. Although there are limitations to the existing generic patient reported outcomes measures, a combination of these together with a disease-specific questionnaire, and physical measures of active and passive individual joint Range of movement (ROM), grip and sensibility using standardised protocols should be used for future outcomes studies. As Dupuytren’s disease tends to recur following treatment as well as extend to involve other areas of the hand, follow-up times should be standardised and designed to capture both short and long term outcomes.

## Background

Dupuytren's disease (DD) is a common fibroproliferative disorder of the hand affecting 4-6% of the population in northern Europe [1]. It is characterized by the progressive thickening and shortening of the palmar fascia, resulting in the formation of cords, flexion deformities of the digits, and hence loss of range of motion, particularly functional extension [2]. DD often restricts a patient’s ability to undertake daily activities of living, depending on the location, extent and severity of disease. The deterioration in hand function is the main motivator for patients seeking treatment [3].

DD is progressive, advancing at varying rates, and may occur bilaterally. It can also recur in the same digit following treatment or appear at another site in the hand (disease extension). Furthermore, the effects of recurrence are not uniform and some patients may opt to have further intervention. Therefore, the development of a uniform set of measures to accurately assess functional impairment has proved challenging.

Most patients with early disease, i.e. before the development of digital contractures, are left untreated, although radiation [4] and steroid injections [5] have been used. Currently, the mainstay of treatment for patients with established flexion deformities is surgery in combination with hand therapy. The aim of surgical procedures for DD is to preserve and, if possible, improve hand function. Surgery comprises either the division (fasciotomy) or removal (fasciectomy or dermofasciectomy) of the diseased tissue. Needle fasciotomy divides the cord in a minimally invasive manner. However, recurrence is common, with one study [6] reporting a rate of 84.9% at 5 years following needle fasciotomy compared with 20.9% following limited surgical fasciectomy. Discontinuity of the cords may also be achieved enzymatically, with 77% of metacarpophalangeal joints and 40% of proximal interphalangeal joints being corrected to within 5 degrees of full extension [7]. However, like needle fasciotomy, recurrence rates are high, affecting 56% of PIP joints and 27% of MCP joints at 3 years [8].

### The need for a systematic review on outcomes used in Dupuytren’s disease

The optimal treatment in DD remains controversial, in large part due to the lack of evidence based on good quality clinical outcomes studies. Not only is there a paucity of research reporting functional outcomes data for DD but also a lack of consensus on the parameters that should be used. Traditionally, the evaluation of treatment success for DD has largely been determined by clinical examination and physical measures, although improvement in physical measures does not necessarily reflect function [9]. Functional outcome measures specifically assess the consequences of impairment in daily activities. A recent systematic review of efficacy and safety of DD surgery in European patients noted that the commonest outcome measure was improvement in mean joint contracture [10]. However, hand function outcomes were not included in this review. The scarcity of studies reporting functional outcomes following treatment for DD has previously been highlighted [11] and noted in a review of surgical treatments for primary DD [12]. More recent studies have monitored functional outcomes using patient reported outcome measures (PROMs), which have been recognised as an important measure of the effectiveness of care from the patient’s perspective [13].

The aim of this review is to identify and determine the relevance and efficacy of outcomes measures used to assess change in hand function following treatment DD by surgery, needle fasciotomy or collagenase injection. This will provide an evidence base to inform the selection of appropriate measures for future research on the management of patients with DD.

## Methods

### Search strategy

The search strategy and search terms were based on a Participants, Intervention, Comparison, Outcomes and Study design (PICOS) [14] (Additional file 1: Table 1). We included studies published over the last 20 years in the English language of adults who had surgery, needle fasciotomy or collagenase injection for primary or recurrent DD where outcomes were monitored using standardised PROMs, functional tests or physical measures as opposed to recording unsubstantiated improvement. Functional outcomes measures reported in randomised and non-randomised controlled clinical trials, prospective and retrospective case series were included. Non-invasive interventions including radiotherapy, steroid injection alone, splinting and skeletal traction were also excluded, as were case studies, conference abstracts and letters. There was no restriction regarding the time that patients were monitored post-intervention.

### Search methods for identification of studies

A literature search was performed in July 2012 using subject heading and free-text terms. Three databases, Ovid Medline, Embase and CINAHL (via EBSCOHost) were searched from 1992 to July 2012. PubMed was also searched from September 2010 - July 2012 to capture electronic publication ahead of print studies not included in Ovid Medline. Searches were limited to studies in English. Search results were imported into an EndNote library and 616 publications were identified for title/abstract review after removal of duplicates.

Two authors (CB, ALP) individually screened the studies using the study eligibility tool to identify studies for inclusion with information gained from the electronic databases (minimum title). Where necessary, consensus was achieved by reviewing the full text. Five hundred and twenty five studies were excluded, resulting in 90 articles meeting the inclusion criteria. Following detailed assessment of the included articles, one further publication was identified from their reference lists. The study selection process is summarised in Additional file 2: Figure 1.

### Data collection and analysis

Data on intervention, population and outcomes were collected and tabulated on a Microsoft Excel (Microsoft, Seattle) spreadsheet. Amalgamation of data for statistical analysis was not possible due to the heterogeneity of the studies. Therefore, description of the data is presented to illustrate the variety of functional outcome measures used and the quality of the data. Where available, numerical data are presented as means with range and standard deviation.

## Results

### Clinical study design

Of the 91 included studies, 66 pertained to surgery, 16 to needle fasciotomy and 9 to collagenase injection. All 9 publications reporting the outcomes of collagenase injection were randomised controlled trials or prospective studies. Many studies reporting surgery (n=29, 43.3%) were retrospective reviews. Eighteen publications reporting outcomes for surgery and 4 needle fasciotomy studies were prospective. In 17 studies (18.7%) it was not possible to establish whether they were prospective or retrospective in design and were categorised as ‘unclear’. The functional outcomes used for each of the three interventions is summarised in Additional file 3: Table 2.

### Patient reported outcome measures (PROMs)

#### Measures of function

**Figure 1:**
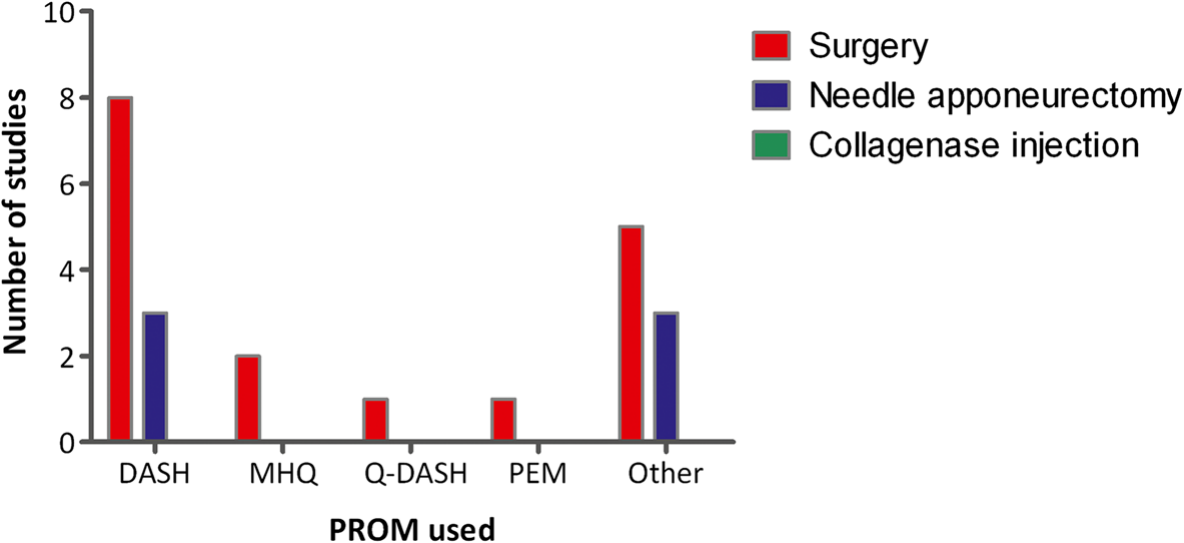
**Use of function patient reported outcome measures according to intervention surgery**, **needle fasciotomy and collagenase injection.**

Twenty-two studies included patient reported data using function PROMs with 13 using validated region-specific questionnaires: the Disability of Arm, Shoulder and Hand (DASH) (n=11), Quick-DASH (n=1) and the Patient Evaluation Measure (PEM) (n=1). Two used the Michigan Hand Outcomes Questionnaire (MHQ) in addition to the DASH. A recently developed disease-specific questionnaire, Unité Rhumatologique de Affections de la Main scale (URAM), was utilised by one study [15] and 8 used other self-report measures such as return to work or self-rated disability. A further 5 reported the use of function PROMs but did not present the results [16]–[20]. The use of PROMs is summarised in Figure 1.

The DASH questionnaire [21] was the most frequently used function PROM (11 studies) but only 6 studies presented calculated pre-and post-treatment data [22]–[27] (Table 1).

**Table 1:** **Number of study subjects and timing of post treatment assessment in studies using DASH and reporting pre**- **and post**-**operative data**)

**Author**	**No. study subjects**	**Assessment schedule (range)**	**Pre**-**op**	**Post**-**op (final)**
			**Mean (range/ SD)**	**Mean (SD)**
Engstrand et al.	60	Pre and 3 months	17 (7–28)	7 (3–12 interquartiles)
Herweijer*	46	Pre and mean 10 (7 to 13) months	12.1 (12.9)	6.6 (8.8)
Jerosch-Herold,	148	Pre, 3, 6 and 12 months	16.4 (15.1) Splint group	7 (14.6) Splint group
			15.4 (13.2) No Splint group	6 (9.2) No Splint group
Johnston et al. *	17 pre-op and 16 post-op	Pre, 3 and mean of 14 (11 to 16) months	24 (20)	8(8)
Skoff,	30 (cohort of 10 and 20 subjects respectively)	Pre and average of 42 (37 to 48) months for cohort of 10 subjects. Pre and average 32 (range 24 to 36) months for cohort of 20 subjects.	37 ‘mild disability’ raw data	30 ‘no self-perceived disability’ raw data
Sobierajska	35	Pre, 1 and 3 months	17.5 (14.88)	15.02 (15.25)
Van Rijssen (a),	50 PNF	Pre, 1,2,3,4 and 5 weeks	16 (14) PNF group	9 PNF group
	47 LF		14 (12) LF group	16 LF group
Zyluk	54	Pre, 3 and 12 months	54 (30 to 103) raw data	32 (30 to 104) raw data

*Also reported MHQ data in same scheduling.

Number of study subjects and timing of post treatment assessment in studies using DASH and reporting pre- and post-operative data.

The DASH score is calculated using the formula ((sum of n responses) -1)/nx25), where n represents the number of completed items, resulting in an aggregate calculated score ranging from 0 to 100, with a higher score indicating a greater level of disability [21]. Mean pre-treatment values ranged from 12.1 (SD 2.9) [23] to 24 (SD 20) [25] and post-treatment from 3.44 (range 0 to 52.5) [28] to 8 (SD 8) [25]. It should be noted that a further two studies presented uncalculated or ‘raw’ DASH scores evidenced by reporting a score in excess of 100 [29] or interpreting a score of 30 as ‘no self-perceived disability’ [30] and were excluded as presentation of the data in this way precluded comparison with other studies.

Two studies additionally used the Michigan Hand Questionnaire (MHQ) [23,25]. The MHQ [31] is scored using an algorithm described in the MHQ codebook [32]. Scores can range from 0–100 for each hand, with higher values representing better function, except for pain, where a higher score indicates more pain. Both studies reported significantly improved global MHQ scores compared to pre-operative scores.

One study utilised the Patient Evaluation Measure (PEM) [33]. The PEM is a validated PROM [34] comprising 3 sections combining hand function and satisfaction with outcome and treatment by asking patients to compare their current versus pre-treatment hand function. The questionnaire is not administered before treatment. The score is calculated by summing the value of the 14 questions in parts 2 and 3 of the PEM and expressed as a percentage of the maximum possible score, with 0% representing a ‘normal’ hand. The study assessed hand function and disability in a retrospective postal audit at a mean follow-up of 27 months (SD 8, range not given) and found a correlation with patient assessed deformity. PEM scores ranged from 9 (SD14, range not given) for patients with no deformity to 57 (SD 25, range not given) for severe deformities at both the MCP and PIP joints.

Mean final follow-up times for function PROMs ranged from 3 months [22,35] to 4.5 years [36]. Final follow up times for studies recording pre- and post-operative DASH scores varied between 5 weeks and 48 months.

#### Patient satisfaction

Eighteen studies used patient satisfaction as an outcome, with no single method used consistently. These included a visual analogue scale of satisfaction [27,37]–[39], satisfaction with treatment [40,41], as well as various descriptive responses such as ‘very pleased, pleased or disappointed [42], or ‘rating of overall clinical success’ [39]. Follow-up times ranged from 1 month [43] to median of 4 years (range 1 – 15) [41].

### Tests of function

Two studies [44,45] utilised the Sollerman hand function test. The Sollerman test comprises 20 unilateral and bilateral hand tasks performed using standardised hand grip patterns intended to reflect normal function in activities of daily living [46]. Performance is graded by the examiner according to the quality of the grip and the time taken to perform each task. The scores range from 0 to 80, with normative values of 80 for the dominant hand and 77 to 80 for the non-dominant hand. Scores were 72.8 (range not reported) pre-treatment and 78.03 (range not reported) 6 months post-treatment [44]. A second study at the same centre reported scores of 71 (range 62 to 80) pre-treatment and 77 (range 66 to 80) 6 months post-treatment [45]. Neither publication reported whether the improvement was statistically significant.

### Physical measures

#### ROM

Range of movement was the most frequent measure and was reported in all 91 studies. However, the methods of recording range of movement varied, with 17 different descriptors. Table 2 summarises the range of motion reporting of all studies. Techniques most commonly used in order of frequency included measurement of flexion contracture (n=24), joint extension deficit (n=18), Tubiana system which grades the DD contracture into one of four stages based on the combined angles of the MCP and PIP joints (n=16), active flexion and extension deficit (n=16) and the use of author defined categories such as ‘improved’ or graded according to degree of contracture defined by the author (n=14). Twelve other methods were used in 35 studies (Table 2). Follow-up times post treatment ranged from 2 weeks [27] up to 17 years [47].

**Table 2:** **Range of motion reporting**: **all studies n**=**91 **(**NB**. **29 studies reported more than 1 method**)

**Range of motion reporting**	**n**=**91**	
**Description**	**Study reference number**	**Frequency**
Flexion contracture	[16,17,20,30,37,39,48]–[65]	24
Joint extension deficit	[17,18,36,38,45,55,66]–[77]	18
Active flexion and extension of joint	[7,20,25,30,51,53,66,70],[78]–[85]	16
Tubiana Grading system	[15,27,43,60,67,71,75,79],[86]–[93]	16
Author defined category	[19,33,40,44,47,64,77,78],[94]–[99]	14
Passive extension deficit	[6,7,27,43,61,78,85]–[87,89,90,100]	12
Fixed flexion deformity	[94,97,101]–[104]	6
Total Active Motion	[23,24,35,41,66,70]	6
Flexion deformity	[99,105]–[107]	4
Composite flexion	[27,86,87]	3
Flexion deficit	[27,71]	2
Extension contracture	[29,108]	2
Total lack of active extension	[26,35]	2
Percentage change	[42,101]	2
Total digital extension	[22]	1
Total active extension deficit	[109]	1
Total lack of active flexion	[35]	1

Additional file 1: Table 1: Search strategy and search terms using PICOS analysis.

#### Grip and Pinch strength

Measurements of grip strength or pinch grip were presented in 11 studies but only 3 reported pre- and post-intervention data [23,25,29] and 3 gave a descriptor of post-treatment outcomes, for example ‘no significant change or deterioration’ [20,48,66]. Follow-up times ranged from a median of 81 days [66] to a mean of 3.5 years [28].

#### Sensibility

Of 11 studies that reported sensibility testing, only one presented pre- and post-operative data [23]. Sensibility was tested using two-point discrimination (2PD) in 6 studies, Semmes Weinstein Monofilaments (SWM) by 1 group and assessment of light touch by another using the fingertip. No collagenase injection studies recorded sensibility. Three studies reported testing sensibility but data were not presented [16,86,87]. Follow-up times after treatment ranged from means of 10 months [23] to 4.4 years [105].

**Figure 2:**
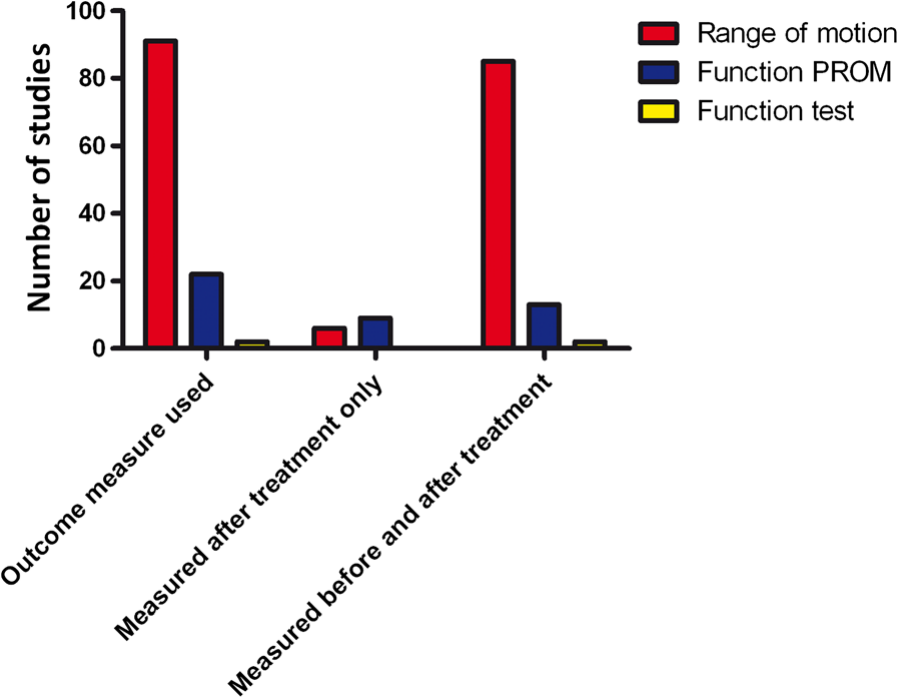
Frequency of outcome measures used and number of studies that measured before and after treatment.

Figure 2 summarises the frequency of the various functional outcomes used before and after each type of intervention.

## Discussion

Our review highlights the heterogeneity of outcome measures used for reporting the results of treatments for DD and the challenges faced when attempting to interpret data to determine best practice. Furthermore, many studies reported in insufficient detail how data were collected. Inconsistencies of outcome measures used and follow-up times have been highlighted previously [12].

The importance of utilising a set of commonly accepted outcome measures that are validated is paramount so that the natural history of the disease as well as treatment success can be monitored at both the patient and population level to enable meaningful comparisons between treatment modalities. Therefore, there is an urgent need for a common consensus regarding the reporting of outcomes data collected in high quality studies.

### Patient reported outcome measures

PROMs, although subjective, are critical measures of treatment efficacy as patient perceived benefit is the ultimate goal of treatment. They should, therefore, supplement data derived from physical measures as they provide the context of the impact on function for the individual. PROMs including functional measures have gained prominence with the current emphasis on patient centred clinical practice. Yet, no publication of collagenase injections used PROMs as an outcome and only 15 publications reporting surgical outcomes used function PROMs. Only one RCT reported outcomes using function PROMs [24].

The DASH questionnaire is a 30-item self-rated region-specific disability and symptom scale outcome measure. It was the most commonly used function outcome measure but may lack the sensitivity to detect significant improvement following surgical or injection treatment for DD due to a ‘flooring effect’, that is relatively low pre-treatment scores resulting in a reduced potential for improvement [24]. It is difficult to be certain whether this represents a genuine problem as only 6 studies reported pre-treatment DASH scores, although all showed comparatively low values. The normal mean value for the DASH questionnaire reported by the American Academy of Orthopaedic Surgeons [110] was 10.10 (+/- 14.68 SD). All publications reporting pre- and post-operative data showed a reduction in post-operative scores, indicating an improvement in function compared with pre-operative levels, but this reached significance in only two studies [22,23]. A difference of 15 points is considered to be the minimal clinically important difference (MCID) indicating an improvement [111]. However, the exact figure is controversial and may vary according to the upper limb disorder being considered. No studies achieved a MCID equal to or more than 15 points and only two studies showed a MCID of 10 points [22,25], which may in part be attributed to the relatively low pre-treatment scores. Additionally, caution is advised when applying MCIDs due to difficulties with variation over time of in the patients’ perception of their disability [112]. Thus, whilst the evidence for assessing the outcomes of interventions for DD using DASH indicates that it may be useful, further work is necessary to determine the level of change that is considered meaningful.

The Michigan Hand Outcome Questionnaire (MHQ) is a 37-item region-specific outcome measure that includes 6 sub-scales of activities of daily living, hand function, pain, cosmesis, patient satisfaction and overall function [31]. The MHQ has been shown to detect change in function following surgery for DD [23,25] and to correlate with changes in fixed flexion deformity [25]. The MHQ focuses on the hand as compared with the DASH, which assesses entire upper limb function. Furthermore, unlike DASH, the MHQ assesses the functional impact on each hand separately; this may be especially relevant to conditions such as DD that can affect both hands to varying extents. It also includes questions that may be of greater relevance for people with DD. For instance, aesthetics are a construct in the MHQ not included in the DASH and may be relevant for some patients with DD, for example when shaking hands [3] or when presenting the hand with the palm uppermost as when receiving coins.

Whilst the MHQ may appear to be more suitable than the DASH to assess DD outcomes it is lengthy, comprising 74 questions, and may not always be completed. Short versions of the DASH and MHQ have recently become available. The Quick-DASH was used by one study [66] but it is not clear if it is prone to a flooring effect when used for DD. The use of the short MHQ has not yet been reported in outcomes studies for DD. Pain, which is included in both the DASH and MHQ, is seldom reported by people with DD [113] and may reduce the sensitivity of both tools.

The PEM has been shown to be sensitive to change when used for patients with scaphoid fractures or carpal tunnel syndrome [114]. In a study of 100 patients with various conditions affecting the hand [115], including 15 who had DD, patients were able to complete it more rapidly than the DASH or MHQ. A sub-analysis of the results for the patients DD has not been published. Therefore, it is not possible to assess the sensitivity to change in the context of DD.

While there are a number of other hand function questionnaires, these have either been validated for use with specific conditions such as the ABILHAND for rheumatoid arthritis, chronic stroke and systemic sclerosis [116] or do not assess the area affected by DD, such the Patient Rated Wrist Evaluation (PWRE) [117]. Disease-specific hand function questionnaires are generally considered to be desirable as they focus on activities that specifically affect the study population, usually having been developed with patient participation. An example that has been shown to be sufficiently sensitive to change is the Boston questionnaire for carpal tunnel syndrome [118]. Recently the Unité Rhumatologique de Affections de la Main scale (URAM) questionnaire [15] and Southampton Dupuytren’s Scoring System [119] have been developed as disease specific questionnaires, but have not yet been validated by other investigators.

An alternative approach is to ask individual patients to identify their most restricted activities and when applied to DD, significant improvement in median scores were reported [22].

#### Patient satisfaction

Methods used to report patient satisfaction varied, with no single measure used consistently. Studies reported overall satisfaction or satisfaction with surgery but did not explore the relationship with hand function. One study of collagenase injection [78] correlated satisfaction with treatment and improved range of motion at 30 days after the last injection.

### Tests of function

Functional tests are used to provide objective ‘measurable’ assessment of function. However, critics question their ability to accurately reflect function due to the strict test conditions. Speed of task completion is used to identify change and does not reflect everyday conditions experienced by the patient. The Sollerman was the only functional test used to date in patients with DD. The 2 studies [44,45] using this measure were undertaken in the same centre in the UK, with both reporting change. However, lack of information on reliability, validity and sensitivity for patients with DD, together with lengthy completion time [120] and the need for cumbersome equipment, may limit widespread use.

### Physical measures

#### Range of movement

Range of motion was the most commonly used outcome measure and was reported by every study. It is sensitive to change and benefits from a high inter-rater reliability for patients with DD [121]. However, the relationship between the degree of flexion contracture and loss of function in patients with DD is not necessarily linear [9,29,122], and the amount of change of motion required to translate into a significant functional improvement is unclear and may vary according to the patient and disease severity prior to treatment.

Although ROM was documented in all studies, the reporting was not standardised, making comparison between publications difficult. While studies may appear to be describing the same measure (joint extension deficit, fixed flexion deformity, flexion deformity, flexion deficit, extension contracture), the lack of a standardised measurement protocol means that this cannot be assumed. For example, many studies did not state how hyperextension was recorded, a common finding at the distal interphalangeal joint or as a compensatory movement at the metacarpophalangeal joint (MCPJ) especially for the little finger in patients with isolated proximal interphalangeal joint (PIPJ) contractures. Fixed deformities should be recorded separately and whether the apparent fixed flexion at the PIPJ can be overcome by flexing the MCPJ should be noted.

The grading system originally described by Tubiana was the joint third most frequently reported measure used for ROM [123]. Clinically it allows easy identification of improvement but not the location of change as it combines the measures of the MCP and PIP joints. Therefore, we suggest that if using the Tubiana grading, it should be supplemented with additional measures to detail the individual joints at which the improvement has been achieved. Similarly, composite ROM measures of finger joints, such as total active motion (TAM), do not permit identification of change at individual joints.

#### Grip

Measurements of pinch and grip strength are important aspects of hand function [124] and have been shown to have a strong correlation with disability [125]. However, only 3 of 10 studies that recorded grip or pinch grip strength presented data before and after treatment. Furthermore, assessment of grip and pinch were often not standardised. Grip strength is best measured using a protocol such as that recommended by the American Society of Hand Therapists. A reliable assessment of grip strength is important to capture clinical changes in interventional studies.

#### Sensibility

Sensibility testing was mainly used to monitor complications such as inadvertent nerve injury [16,87]. Most authors assumed that participants had normal sensibility before intervention, with only one recording pre-treatment values. Although testing using Semmes Weinstein Monofilaments has been shown to be a superior measure of sensibility assessment following nerve injury than static two point discrimination [126], they were used in only one study.

### Limitations of the review

Excluding articles not published in the English language may have led to potential bias and heterogeneity of studies precluded pooling of data for meta-analysis. The inclusion of low quality studies presenting retrospective data may be viewed as a limitation but an exclusive approach would have resulted in an incomplete picture of current methods used.

## Conclusions

Studies to date on the outcomes of treatments for patients with DD are heterogeneous and of relatively poor quality. It is critical to achieve a consensus regarding validated outcome measures to monitor disease progression and treatment success in order to enable comparisons between treatment modalities. Subtle deterioration of hand function with functional adaptation in patients with DD over time makes this challenging. Therefore, it is important to use measures that accurately assess global and local disability in a sensitive and specific manner in order to successfully evaluate any change in hand function as a result of intervention. Change in hand function is best assessed by a combination of physical measures and questionnaires [127]. DD can affect multiple digits of the same hand but can also occur bilaterally so comparison with the contralateral hand is not always possible. Inclusion of more than one hand or finger per patient and patient drop out resulting in varying numbers at follow-up represent possible sources of bias.

Based on our findings we recommended the use of a region specific questionnaire such as the MHQ and a validated disease specific PROM like the URAM. A useful addition would be designation of tasks important to each patient and indicating on a linear scale the degree of difficulty before and after treatment. Patient satisfaction should be assessed using a valid and reliable questionnaire such as the Picker questionnaire [128] or PEM. Physical measures of joint ROM, grip and sensibility should be included, with ROM measured before and after treatment using a standardised protocol. We recommend recording measurement of active flexion and extension of each joint and including whether an apparent fixed flexion deformity of the PIPJ can be overcome with passive flexion of the MCPJ. Grip strength should be measured using a validated instrument, such as the Jamar dynamometer according to a standardised protocol. Assessment of sensation using Semmes Weinstein Monofilaments should be used to monitor complications.

Hand function may also be compromised by recurrent disease. Scheduling of follow-up assessments may differ between surgery and other less invasive interventions but final follow-up time should be standardised to capture recurrence and understand how this affects function [129]. Following needle fasciotomy and collagenase injection initial improvement in range of motion and satisfaction may compare favourably with surgery in the short-term but recurrence may compromise function and satisfaction in the long-term.

A comprehensive and consistent approach will enable the development of a robust and reliable evidence base upon which to base best clinical practice for the treatment of patients with Dupuytren’s Disease.

## Competing interests

The authors declare that they have no competing interests.

## Authors' contributions

CB, ALP performed the literature search and together with JN analysed the data. All wrote the manuscript. All authors read and approved the final manuscript.

## Pre-publication history

The pre-publication history for this paper can be accessed here:

http://www.biomedcentral.com/1471-2474/14/131/prepub

## Supplementary Material

Additional file 1: Table 1 Search strategy and Search terms using PICOS analysis. Click here for file

Additional file 2: Figure 1 Study selection process flow chart. Click here for file

Additional file 3: Table 2 Functional outcomes reported by intervention; Collagenase injection, needle fasciotomy and surgery [3,6,7,15,16,20,22]–[30,33,35]–[45,47,48,50]–[109]. Click here for file
