# Supplementary material for: Optimal functional outcome measures for assessing treatment for Dupuytren’s disease: a systematic review and recommendations for future practice
Source: BMC Musculoskelet Disord. 2013 Apr 10;14:131. doi: 10.1186/1471-2474-14-131 (PMC3637830; doi:10.1186/1471-2474-14-131)
Supplement: Additional file 1: Table 1 — Search strategy and Search terms using PICOS analysis. [file 1471-2474-14-131-S1.doc]

## Additional table 1

Search strategy and Search terms using PICOS analysis

|  | **Definition** | **Main Search Terms for Ovid Medline Strategy**  **Subject heading (/) and free text terms** |
| --- | --- | --- |
| **Participants** | Persons with Dupuytren’s Disease | Dupuytren’s contracture/ or dupuytren |
| **Intervention** | Surgical treatment including percutaneous or collagenese injection for DD of the hand. | Exp surgical procedures, operative/ or surgor fasciectom or fasciotom or dermofasciectom or open palm or mccash or aponeurectomor aponeurotom  Injections/ or injections, Intralesional/ or injector collagenases/ or microbial collagenases/ or collagenase |
| **Comparisons** | Not applicable |  |
| **Outcomes** | Patient Reported Outcome Measures and Functional Tests  Physical measures, satisfaction, quality of life | Exp treatment outcome/ or outcome assessment (healthcare)/ or outcome or hand or exp hand strength/ or exp range of motion, articular/ or range of motion or grip strength or follow-up studies/ or quality of life/ or questionnaires/ or disability evaluation/ or disab or disab or efficac exp patient satisfaction/ |
| **Study design** | All Included:-  Screen search results manually to include RCT’s and non-randomised controlled clinical trials, prospective and retrospective case series.  Excluded:-  Case studies and conference papers. | As described in the study |
