# Supplementary material for: Optimal functional outcome measures for assessing treatment for Dupuytren’s disease: a systematic review and recommendations for future practice
Source: BMC Musculoskelet Disord. 2013 Apr 10;14:131. doi: 10.1186/1471-2474-14-131 (PMC3637830; doi:10.1186/1471-2474-14-131)
Supplement: Additional file 2: Figure 1 — Study selection process flow chart. [file 1471-2474-14-131-S2.docx]

**Additional Figure 1** Study selection process flow chart

Study selection process Flow chart

**1100 hits using search strategy**

Ovid medline =388 Ovid embase =248

CINAHL (EBSCO) =54 PubMed Ovid Pre-medline (2011-12) =110

Prospective = 2

RCT’s

= 7

RCT’s

= 3

Retrosp-ective = 5

Retrosp-ective =29

Unclear

= 4

Prospective = 4

Combined methods = 3

Unclear = 13

Prospective = 18

RCT’s = 3

**Surgery = 66**

**91**

**484 Excluded**

**525 Excluded**

**Needle fasciotomy = 16**

**Collagenase injection = 9**

CB & ALP **screened using study eligibility tool** (appendix 2), +/- discussion for agreement

**616 Identified by title/abstract following** **removal of** duplicates
