# Supplementary material for: Optimal functional outcome measures for assessing treatment for Dupuytren’s disease: a systematic review and recommendations for future practice
Source: BMC Musculoskelet Disord. 2013 Apr 10;14:131. doi: 10.1186/1471-2474-14-131 (PMC3637830; doi:10.1186/1471-2474-14-131)
Supplement: Additional file 3: Table 2 — Functional outcomes reported by intervention; Collagenase injection, needle fasciotomy and surgery [3,6,7,15,16,20,22]–[30,33,35]–[45,47,48,50]–[109]. [file 1471-2474-14-131-S3.docx]

***Additional Table 2: functional outcomes reported by intervention; Collagenase injection, needle fasciotomy and surgery***

Functional outcomes reported by intervention: Collagenase injection, needle fasciotomy and surgery,

|  | **Collagenase Injection (n=9)** | | **Needle fasciotomy (n=16)** | | **Surgery (n=66)** | |
| --- | --- | --- | --- | --- | --- | --- |
|  | **Study reference number** | **Frequency** | **Study reference number** | **Frequency** | **Study reference number** | **Frequency** |
| **Patient Reported Outcome Measure** |  |  |  |  |  |  |
| DASH |  |  | [29, 30, 39] | 3 | [24-28, 31, 32, 37] | 8 |
| Michigan Hand Outcomes Questionnaire |  |  |  |  | [25, 27] | 2 |
| Quick-DASH |  |  |  |  | [51] | 1 |
| PEM |  |  |  |  | [35] | 1 |
| URAM |  |  | [17] | 1 |  |  |
| Other |  |  | [42, 45] | 2 | [24, 38, 100, 101, 105, 122] | 6 |
| **Functional test** |  |  |  |  |  |  |
| Sollerman |  |  |  |  | [46, 47] | 2 |
| **Physical measures** |  |  |  |  |  |  |
| Range of motion | [8, 41, 50, 67, 78, 79, 81, 124, 125] | 9 | [6, 7, 17, 29, 39, 42, 45, 52, 53, 88, 89, 105, 106, 108, 113, 118] | 16 | [18-22, 24-28, 30-32, 35, 37, 38, 40, 43, 44, 46, 47, 49, 51, 54, 80, 82-87, 90-104, 107, 109-112, 114-117, 119-123, 126-131] | 66 |
| Sensibility |  |  | [29, 52, 53] | 3 | [25, 30, 43, 54, 111, 112, 114] | 7 |
| Grip and Pinch | [8, 79] | 2 | [39] | 1 | [22, 25, 27, 30, 31, 37, 51] | 7 |
| Satisfaction | [67, 81] | 2 | [29, 39, 42, 45, 52] | 5 | [32, 40, 43, 54, 98, 110] | 6 |
